# Cleaning Up without Messing Up: Maximizing the Benefits of Plastic Clean-Up Technologies through New Regulatory Approaches

**DOI:** 10.1021/acs.est.3c01885

**Published:** 2023-08-28

**Authors:** Jannike Falk-Andersson, Idun Rognerud, Hannah De Frond, Giulia Leone, Rachel Karasik, Zoie Diana, Hanna Dijkstra, Justine Ammendolia, Marcus Eriksen, Ria Utz, Tony R. Walker, Kathinka Fürst

**Affiliations:** † Norwegian Institute for Water Research, Økernveien 94, 0579 Oslo, Norway; ‡ University of Toronto Trash Team, University of Toronto, Toronto, Ontario M5S 1A1, Canada; § Ocean Conservancy, Washington, D.C. 20036, United States; ∥ Ghent University, Research Group Aquatic Ecology, Coupure links 653, 9000, Ghent, Belgium; ⊥ Flanders Marine Institute, (VLIZ), InnovOcean Site, Jacobsenstraat 1, 8400, Ostend, Belgium; # Research Institute for Nature and Forest, Aquatic Management, Havenlaan 88, 1000, Brussels, Belgium; □ Research Foundation − Flanders (FWO), Leuvenseweg 38, 1000, Brussels, Belgium; ■ Nicholas Institute for Energy, Environment & Sustainability, Duke University, Durham, North Carolina 27708, United States; ○ Division of Marine Science and Conservation, Nicholas School of the Environment, Duke University Marine Laboratory, Duke University, Beaufort, North Carolina 27708, United States; ● Integrated Toxicology and Environmental Health, Nicholas School of the Environment, Duke University, Durham, North Carolina 27708, United States; △ Institute for Environmental Studies, Vrije Universiteit, De Boelelaan 1111, Amsterdam, Netherlands; ▲ School for Resource and Environmental Studies, Dalhousie University, Halifax, Nova Scotia B3H 4R2, Canada; ⬡ Faculty of Graduate Studies, Dalhousie University, Halifax, Nova Scotia B3H 4R2, Canada; ⬢ The 5 Gyres Institute, Los Angeles, California 90409, United States; ¶ Sciences Po Paris, 27, rue Saint-Guillaume, 75007, Paris, France; ◆ University of California, Berkeley, Berkeley, California 94720, United States

**Keywords:** plastic pollution, litter, clean-up technology, bycatch, externalities, regulations, added value, plastics treaty

## Abstract

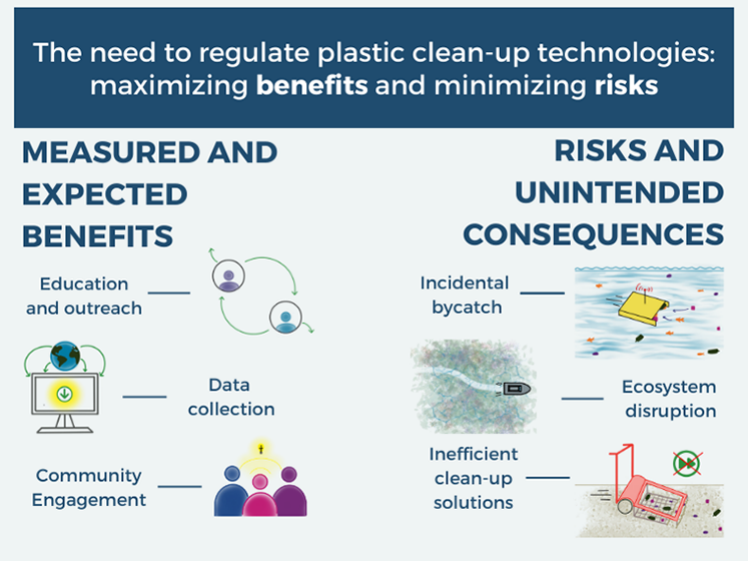

As the global plastics crisis grows, numerous technologies have been invented and implemented to recover plastic pollution from the environment. Although laudable, unregulated clean-up technologies may be inefficient and have unintended negative consequences on ecosystems, for example, through bycatch or removal of organic matter important for ecosystem functions. Despite these concerns, plastic clean-up technologies can play an important role in reducing litter in the environment. As the United Nations Environment Assembly is moving toward an international, legally binding treaty to address plastic pollution by 2024, the implementation of plastic clean-up technologies should be regulated to secure their net benefits and avoid unintended damages. Regulation can require environmental impact assessments and life cycle analysis to be conducted predeployment on a case-by-case basis to determine their effectiveness and impact and secure environmentally sound management. During operations catch-efficiency and bycatch of nonlitter items, as well as waste management of recovered litter, should be documented. Data collection for monitoring, research, and outreach to mitigate plastic pollution is recommended as added value of implementation of clean-up technologies.

## Abstract 2

As clean-up technologies might have unintended negative consequences, we suggest how such technologies can be regulated under the plastics treaty to secure their net benefit.

## 1 Introduction

Plastic pollution is one of the greatest environmental challenges facing the world today, threatening human and environmental health.^1^ Initiatives from local to global levels have been launched to address the issue, including the current United Nations negotiations toward a global plastics treaty.^2−4^ Although upstream actions (e.g., reduction, substitution, and new product designs and business models) are identified as the most cost-efficient solutions to reducing and preventing plastic pollution,^1,4−6^ a combination of management strategies across the entire plastic lifecycle is required for reducing current and future plastic pollution impacts.^7−10^ These include downstream solutions, such as the collection of plastics in the environment. Globally, manual clean-up activities that engage the public and the deployment of emerging technologies expressly designed to address legacy plastic pollution have been implemented in an effort to mitigate the plastics crisis downstream.^11−13^ These combined efforts have contributed to plastic pollution reductions in the environment, direct benefits to ecosystems and communities (Arabi et al., 2020), and additional cobenefits, such as public awareness and the generation of data to inform policy (e.g., Haarr, Pantalos,^14^ EU,^15^ Wyles, Pahl,^16^ Falk-Andersson, Berkhout,^17^ Canada^18^).

**Figure 1:**
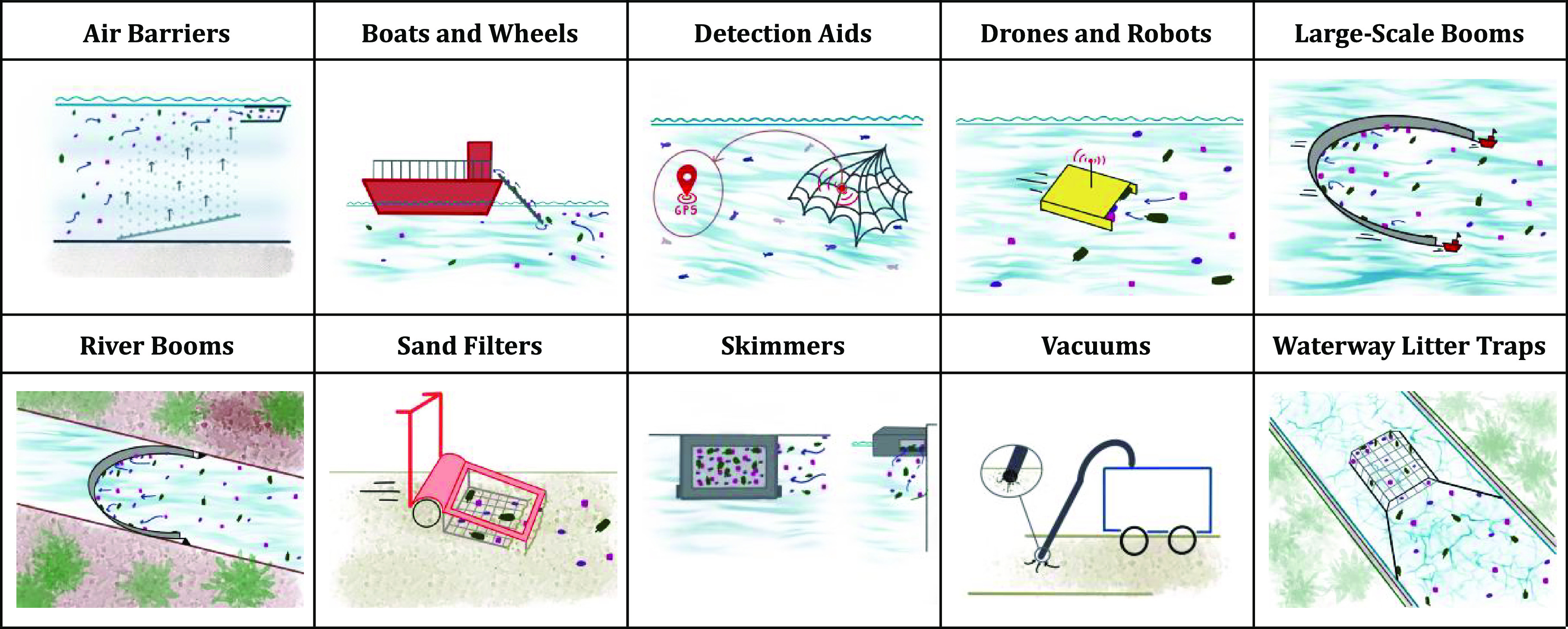
Graphical depictions of categories of plastic clean-up technologies, as classified in Schmaltz, Melvin,^13^ demonstrating the diversity of plastic clean-up technologies that currently exist. These are deployed in various environments, use unique methods, and target different kinds of plastic pollution (all figures are original and developed by authors of this article).

Plastic remediation technologies have been utilized globally, ranging from community-based initiatives to national programs. These technologies can be grouped into two categories: (1) prevention technologies and (2) clean-up technologies.^13^ Plastic prevention technologies are designed to remove plastic and other anthropogenic waste before entering the environment and include filtration systems in wastewater treatment plants and laundry filtration technologies.^13,19,20^ Plastic clean-up technologies have been developed and deployed to remove plastic already present in the environment. The majority of the clean-up technologies cataloged in The Plastic Pollution Prevention and Collection Technology Inventory^13^ as well as in the plastic clean-up and prevention overview^20^ are deployed in aquatic environments. Other environments where plastic clean-up technologies can be employed include sand and soil. Aquatic plastic clean-up technologies include river booms and nets, receptacles, and watercrafts that can be deployed in built, urban, or natural environments.^13,21^ Examples include The Ocean Cleanup,^22^ Mr. Trash Wheel,^23^ and Seabin.^24^ Within plastic clean-up technologies, a variety of designs have been developed to adapt to different environments and contexts^19,20^ ranging from passive to active technologies (Figure 1).

Manual clean-ups have been conducted around the world by paid formal and informal waste collectors as well as volunteers.^25^ However, there may be circumstances where a plastic clean-up technology is more appropriate to increase efficiency, as clean-up technologies can enable access to hard-to-reach litter and mitigate unsafe working conditions. At the same time, clean-up technologies may have unintended consequences and may not always be cost-efficient. Consequently, concerns are raised regarding the cost-efficiency and environmental impact of these clean-up technologies^10,24,26−28^ Concerns are likewise raised that clean-ups distract the public and decision makers from upstream source-reduction strategies,^27^ misrepresenting that plastic pollution can be mitigated solely through downstream approaches. These concerns justify the use of regulatory instruments to oversee the use of clean-up technologies. However, the plastics policy landscape currently lacks explicit guidance or oversight over clean-up technology implementation.

The United Nations Environment Assembly resolution to end plastic pollution specifically refers to the need for the intergovernmental negotiating committee to consider measures to reduce plastic pollution already present in the environment.^29^ The future international instrument may therefore include provisions encouraging countries to include clean-up activities in their national action plans and other implementation measures. Given the current lack of guidance in the international policy landscape, the treaty provisions must be designed to ensure uptake of clean-up technologies does not result in adverse outcomes and instead maximizes positive impacts.

Given the expansion of innovative and novel plastic remediation technologies (to date, over 100 technologies have been recorded^20^) and the diversity of technology types and potential trade-offs, we saw a need to share knowledge and insights to better understand the role of plastic clean-up technologies in combating plastic pollution. In June 2022, a two-session webinar series was organized that brought together diverse stakeholders (i.e., entrepreneurs, nongovernmental organizations, and researchers) in fruitful discussions on the role and contribution of plastic clean-up technologies in reducing plastic pollution. Webinar panelists coalesced around two key messages: (1) regulation of plastic clean-up technologies is needed to ensure a net benefit for the environment and affected communities, and (2) responsible implementation of plastic clean-up technologies can result in cobenefits to society.

**Figure 2:**
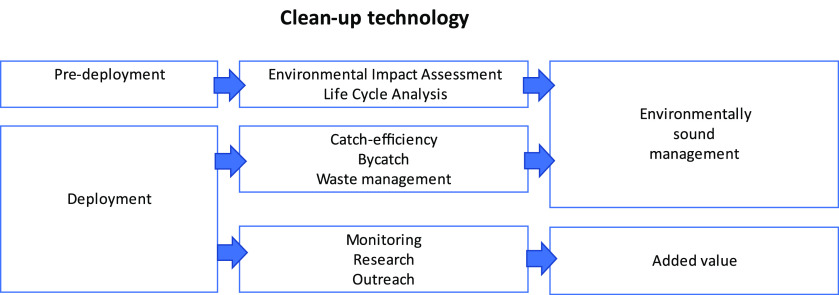
Suggested elements for evaluation of clean-up technologies to secure environmentally sound management and added value. To secure environmentally sound management, EIAs and LCAs should be conducted predeployment, while during deployment the catch-efficiency, bycatch rates, and waste management should be documented. During deployment, collection of data for monitoring and research, as well as implementation of outreach projects, would provide added value of implementing the clean-up technology.

We argue that to maximize cobenefits and mitigate potential negative consequences, the role of plastic clean-up technologies in reducing plastic pollution should be given careful attention in the upcoming international treaty. Here, we elaborate on the key insights from the webinar and clarify the role of plastic clean-up technologies in the solutions landscape to reduce plastic pollution with specific reference to the global plastic treaty currently being negotiated.^2,4,30^ We argue that the treaty should include language and guidance on ensuring clean-ups of existing plastics in an environmentally sound manner (ESM). Elements under the guidelines include predeployment feasibility studies, such as environmental impact assessments (EIA), and/or life cycle analysis (LCA). During deployment, monitoring and reporting of bycatch and litter collected should be conducted to document cost-effectiveness and environmental impact and enable transparency regarding waste management and final fate of collected litter to secure ESM. Additionally, data collection on amounts and sources for monitoring, research, education, and outreach would provide added value (Figure 2).

## 2 Securing Environmentally Sound Management

In international environmental law, the ESM is commonly used to signify that measures will need safeguards to prevent negative environmental externalities. Two examples are the Basel Convention and the Minamata Convention. However, there is no single agreed upon definition of what ESM entails.^31^ Rather, ESM is understood as a “broad policy concept that is implemented in various ways by different countries, organizations and stakeholders”.^32^ In the context of the Basel Convention, ESM implementation is guided by technical guidelines, toolkits, and frameworks.^33^ The Framework for ESM of wastes, adopted at COP11 of the Basel Convention, identified the following elements as needed to be considered: regulatory matters, facility-related matters, waste-related matters, resource and process efficiency, environmental protection, occupational safety and health, organizational matters, transparency, and innovation, research and development.^34^ Such an integrated ESM approach may also be relevant for clean-up technologies. In the next sections, we identify the challenges and opportunities that such technologies represent and the evaluations and documentation that should be conducted predeployment and during deployment.

### 2.1 Predeployment Evaluations

Although the removal of plastics from the environment using clean-up technologies has ecological benefits, negative environmental impacts and implementation costs should also be considered. Depending on the scale and target plastic size, clean-up technologies may impact multiple levels of biological organization, from microbiomes to individual organisms and sessile or floating habitats.^24,26,27,35,36^ For example, deployment of technology and personnel to clean up nurdles on the coast of Sri Lanka was found to cause increased coastal erosion.^37^ Additionally, some studies have reported a high occurrence of organic matter (e.g., algae, seaweed) when sampling plastics collected from clean-up technologies.^24,38,39^ Organic matter has important ecosystem functions. For example, floating mats of *Saragassum spp.* macroalgae are classified as essential fish habitats in the marine environment,^40^ and organic matter is crucial for sustaining ecosystems within and downstream of rivers.^41−43^ Thus, implementing plastic clean-up technologies may pose negative ecological risks. Ecological harm may be reduced through technological innovation (e.g., bycatch reduction analogy from fisheries as described in Falk-Andersson, Larsen Haarr^26^) or implementing the technology where and when the plastic load is high,^44^ the risk of harming ecosystems is low, and the benefit of preventing plastics from reaching vulnerable ecosystems is high.^26,45^

The socioeconomic context in which clean-up technologies will be implemented is also key. Without formal regulatory mechanisms, the deployment of technologies can harm communities already disproportionately burdened by the plastics crisis. For example, the clean-up technology Sweepy Hydro was donated to the Sri Lankan government after the containership X-Press Pearl released 1,680 tonnes of plastic nurdles.^37^ This was an imperfect solution that further burdened the community affected by the spill, as the nurdles extended into the substrate, while the Sweepy Hydros could be used only at the surface. The devices were also vulnerable to clogging with wet sand, which made them ineffective in coastal environments. Finally, as Sri Lanka is facing an economic downturn and fiscal crisis, both spare parts and fuel for the machines are in short supply. Ultimately, manual cleaning turned out to be more cost-efficient in this case.^46^

While plastic can generate value within the waste stream, recovery of plastic litter is generally associated with extra waste disposal cost,^6^ which must be carried by those initiating clean-ups. However, this responsibility is not always clear and is also a potential issue for litter collected at high seas outside of national jurisdiction. Waste disposal costs include sorting, cleaning, transportation, and processing, with any positive revenue from recovered litter being dependent on a number of factors, including the market for the recycled products.^47^ Separation of collected plastics from organic matter may be time-consuming if the ratio of plastics to organics collected is low.^24^ Correct sorting of the recovered litter may require specific expertise as its composition may be highly complex.^47^ Recycling facilities may not be available locally, with transportation for recycling adding environmental and economic costs and a risk of plastics becoming mismanaged in import countries.^47,48^ At present, the quality of recovered ocean plastics is often too low to be accepted for recycling,^19,46^ and recycled ocean plastics have been found to score lower on a range of functional material tests^49^ and environmental indicators in life cycle assessments (LCA).^50^ Both the socio-economic context and the recovered plastic’s quality influence the feasibility and cost-efficiency of using clean-up technology, as well as the destiny of recovered plastics in waste management.

Before deployment we recommend that guidelines for ESM clean-ups include feasibility studies (EIAs and LCA) to evaluate the cost-efficiency of the technologies, the maintenance and management of technologies over time, availability of infrastructure for waste management, and the environmental impact of deployment of the technology. Such assessments should evaluate different clean-up methods against each other, including manual clean-ups, in different contexts to ensure that the best solutions are chosen according to the local context. This would secure the optimal use of society’s resources in reducing plastic pollution and avoid unintended negative consequences.

Development projects that may have positive and negative environmental impacts are often subject to EIA regulations in many jurisdictions.^51^ When conducted properly, EIAs require science-based evidence to help inform decision-making on major projects to reduce, mitigate, and disclose negative environmental impacts. Although some companies have conducted EIAs of their technologies,^52^ there are no standardized national or international regulatory requirements guiding EIAs for plastic clean-up technologies. To successfully conduct an EIA on plastic clean-up technologies, the parameters that influence the chances of biota, organic matter, or plastic being collected must be determined, and impacts on the local ecosystems assessed. The latter should include identification of vulnerable species in time and space that can conflict with clean-ups, for example through negative impacts on breeding or nesting. All these factors will depend on the location, time, and type of technology deployed^26,35,53^ and how recovered plastic is managed after collection. While plastic clean-up technologies are most often deployed in aquatic environments, EIAs and other biophysical assessment tools should consider and compare the impact or benefit of the deployment of these technologies in various environments on site specific or case by case basis, as appropriate. For example, well-understood EIAs typically propose scenarios that include alternatives or no action at all. In the case of clean-up technologies, various devices may perform better than others in some scenarios, and in other cases, not deploying the device or technology at all may be considered the best option.

LCAs of the technology deployed will permit better cost-benefit analyses and help determine the best mitigation strategy for a particular environmental compartment, litter density, and socio-economic capacity. Both floating litter and seafloor clean-ups have extremely expensive capital costs that increase with depth and the upscaling needed to significantly reduce legacy plastics,^27,28^ and as plastic density decreases the cost/benefit may change quickly.^26^ An LCA should include an economic assessment, including capital costs, operating costs (e.g., fuel, repair, maintenance), staff requirements, and installation and extraction costs. The technical and financial capacity to apply and maintain clean-up technologies should also be evaluated. Furthermore, the LCA should include a risk assessment that also evaluates impacts from malfunction of the technology, such as fuel spills, fire risk, and shipping and navigation hazards from lost equipment. While considerations of EIA, LCA, and risk assessment have been proposed here, there is no one single biophysical assessment tool that is recommended. Instead, clean-up technologies should be assessed holistically and on a site-specific basis. For example, if accurate predictions can be made for a technology that has not been deployed yet, then an EIA approach would be well suited. However, if a technology has already been deployed but the incidence of mortalities, injuries, or entanglement of species are assessed against other criteria such as population stability and species conservation status, then the risk of deploying the technology may be considered unacceptable based on biodiversity considerations.

### 2.2 Assessments during Deployment

#### 2.2.1 Catch-Efficiency and Bycatch

Studies have shown that the cost-efficiency of clean-up technologies depends on the type of environment, spatiotemporal litter density, and accessibility. In open oceans and on the ocean floor, cost-efficiency of clean-up technologies is low, while in some rivers and coastal areas with litter hotspots, cost-efficiency may be higher.^10,26,27^ Although recent studies are aiming to unravel the effectiveness of certain clean-up technologies,^54,55^ a lack of data on spatiotemporal litter density as well as capital and maintenance costs limits the ability to clearly evaluate their cost-efficiency.^26,56^ For example, the estimated investment, operational and management costs are 1.24–1.55 USD/kg plastics for Seabins and 22.5–30.1 USD/kg plastics for booms.^6^ But this assumes that plastics represent 80–90% of the catches. The cost of cleaning up 25 tons of litter manually at an isolated island of the Seychelles archipelago was about 8.83 USD/kg plastics.^57^ This cost is expected to be substantially lower in or closer to urban areas. To help markedly reduce plastics already in the environment, implementation of clean-up technologies often needs to be scaled up. Parker-Jurd, Smith^24^ calculated that 500 Seabins were needed to keep a marina of 25,000 m^2^ clean, while Hohn, Acevedo-Trejos^10^ found that even 200 oceanic clean-up devices from The Ocean Cleanup would only have a modest impact on floating ocean plastics globally given the current plastic production trajectory. With upscaling of deployment, the risk to the ecosystems described above also increases.

Studies have attempted to standardize catch per unit effort (CPUE) across different plastic clean-up technologies, but a lack of data limits these efforts (e.g., Falk-Andersson, Larsen Haarr^26^). Furthermore, bycatch is generally not documented.^26^ As an example, The International Trash Trap Network has developed a protocol for collection of data that can be used to estimate litter capture rates (weight/day or hour) and document the occurrence of bycatch. However, documentation of flow rates, specific water body information, and the type of bycatch is not mandatory.^58^ Nationally and regionally established litter monitoring protocols do not accommodate for recording catch-rates and bycatch of nonlitter items.^26^

Harmonization efforts to standardize calculation of catch rates and bycatch need to be strengthened to allow for CPUE comparisons across technologies and with manual cleaning. Such data would support feasibility studies prior to deployment of clean-up technologies as well as during implementation and could also feed into environmental monitoring schemes under the treaty. Harmonization should include standardization of data collection across the multiple clean-up technologies applied, as they differ considerably in their mechanisms for plastic pollution capture, which affects the representativity of items caught.^13,24,35^ It is important that selectivity (e.g., size range of plastic debris captured) is documented and that data collection methods and reporting metrics are harmonized.^53,59,60^ Counts data are generally used in plastics monitoring and would be relevant in this context too. Most clean-up technologies targeting macroplastics are unlikely to capture the very large items that account for the large differences in identifying the main sources of litter when using counts as compared to weights.^61,62^ However, for technologies that also capture smaller plastics, both counts and weight data should be recorded as this affects our understanding of the amounts of litter recovered.^24^ Weight data may also be relevant for comparing catch rates of litter and nontarget biota, as plant material may be difficult to count.

Technical guidelines should be developed to define bycatch limits. Bycatch of biota may be inevitable, and in fisheries management, bycatch limits are commonly used for regulating the severity of this type of impact.^63^ Similar regulations may be applied regarding how and when to implement plastic clean-up technologies. For instance, governments could define times when clean-up technologies should not be used due to higher risks of bycatch (e.g., during seasonal spawning or fish migrations). There should also be requirements to design technologies to minimize bycatch. For example, fishing gear has been developed to minimize bycatch rates through the implementation of sorting grids and turtle exclusion devices, which take advantage of the behavioral differences among species.^64−66^ Just as in the fisheries sector, bycatch regulations are not enforceable without monitoring and reporting of catch and discard rates.^67^ There is a need to record and report bycatch items and rates, particularly for vulnerable species,^26^ as a part of clean-up technology reporting requirements. As in fisheries, compliance in terms of reporting data correctly could be a challenge. Independent observers are used in fisheries, and in recent years the use of remote electronic monitoring has also been explored.^68^ Such measures can also be implemented in documenting litter caught by clean-up technologies.

#### 2.2.2 Waste Management

While large amounts of litter have been recovered from the marine environment, there is very little documentation of their destiny.^69^ Circular economy solutions that allow for recovered litter to re-enter the economy should be strived for and may even represent economic opportunities and thereby a cobenefit. Waste collection represents an important livelihood for marginalized communities in low- and middle-income countries,^70^ and collected plastics can be a source of income through repurposing,^57^ energy sources,^71^ or replacement of bitumen in road constructions.^72^ However, the environmental, social, and economic viability of these solutions needs to be carefully assessed and will depend on the country or location of deployment, as there will be differences in factors such as access to customers, favorable regulatory conditions, and waste management infrastructure.^19,73^ Today there are limited economically and environmentally sustainable end-of-life solutions for recovered plastics.^19,50^ Recovered litter represents a diverse mix of materials, with plastics dominating, that is difficult to separate, clean, and recycle.^69,74^ Utilizing waste in energy recovery is an option, as incineration and pyrolysis can use degraded and mixed plastics as feedstock. However, these processes come with economic and environmental challenges, including a contribution to global greenhouse gas emissions and release of atmospheric pollutants in jurisdictions where appropriate incineration facilities are lacking.^75−77^ Relying on such solutions could also lead to a technological lock-in, which does not address the many problems created upstream in the plastics life cycle. In many cases, landfilling may be the best or only option, but this requires that the landfills have high environmental standards to avoid the leakage of chemicals and litter into the environment. To secure the economic viability of these interventions, planning and development of the clean-up technology in parallel with development of business models are recommended.^78^

#### 2.2.3 Ensuring Cobenefits

##### 2.2.3.1 Monitoring and Research

Clean-up initiatives have generated valuable data documenting pollution levels, identifying sources, informing research, guiding upstream mitigation efforts, and monitoring the impact of policies.^26,59,79^ Technologies for knowledge collection, such as mobile applications (i.e., apps) for documentation of amounts and types of litter (e.g., DebrisTracker,^80^ CleanSwell^81^) have allowed for cost-efficient data collection through citizen science. Apps and protocols for litter quantification and identification applied by national monitoring programs (e.g., Fleet, Vlachogianni,^82^ Ospar,^83^ Vighi^84^), could also be used to document plastic captured using clean-up technologies. The University of Toronto Trash Team, for example, uses data on common items collected via Seabins installed along the Toronto Harbourfront to inform local pollution prevention projects,^85^ and data on items collected by MrTrashWheel are available on their Web site.^86^

The development and application of harmonized protocols would facilitate the use of the data from clean-up technology deployment in monitoring and policy advice. The protocols applied should be harmonized with global monitoring efforts (e.g., UNEP,^62^ Vighi,^84^ COBSEA^87^). To reduce the cost of data collection, simplified protocols, such as citizen science protocols, could be suitable for this purpose. Such proposed guidelines can counteract challenges related to securing the quality of citizen-science data.^88,89^ High resolution of some source categories may be needed to secure data that is important for policy interventions.^90^ For example, in the European Union, specific single-use plastic items documented to be abundant on European shorelines have become policy targets.^15^

##### 2.2.3.2 Outreach

Technological solutions have cross-sector enthusiasm and support and can contribute positively to reducing plastic pollution. Several cobenefits of manual clean-ups have been documented that could also apply to clean-up technologies. These include individual and community empowerment, exemplified by community bonding over a shared issue, and individual engagement to change behavior and identify solutions.^28,91^ Groups engaging in clean-ups can become more connected to their culture and community as members work together to protect their local environment and become motivated for advocacy to drive change on a larger scale,^91^ benefits known as “knowledge building”, “culture building”, and “movement building”.^92^

It is important that such cobenefits are maintained to the extent possible with clean-up technology implementation. A study by Maeda, Brščić^93^ found that while observing a human picking up litter made people less inclined to litter, this was not seen when people saw a robot doing the same. Discarding of more litter to the environment due to the perception that the technologies will remove it has also been identified as a risk.^20^ However, some clean-up technologies are designed to engage communities positively. For example, Mr. Trash Wheel in Baltimore Harbor has googly eyes and a social media personality, making it a friendly and beloved character rising to a local celebrity. The aim of this initiative is to build a sense of community pride and engagement around plastics clean-up and environmental stewardship, inspiring people to keep the environment free of plastic.^86,94^ How clean-ups, including employment of clean-up technologies, affect littering behavior is not well studied as indicated by the review of Chaudhary, Polonsky.^95^ Successful public outreach requires resources and logistics to secure participation, high-quality information and engagement materials, the health and safety of participants, and proper waste management for litter recovered (e.g., for clean-up guidelines, see e.g. Marfo,^96^ OC^97^). To encourage critical thinking, these events should also include reflections regarding solutions to the plastic pollution problem, the role of clean-up technologies, their potential negative impacts, if they are appropriate in all contexts, and the scale of the issue in comparison to the scale of plastic collected.

## 3 The Path Forward

The benefits and potential risks of applying clean-up technologies, as well as associated policy recommendations, are summarized in Table 1. Further guidelines and regulations are essential to ensure the beneficial use of plastic clean-up technologies and to minimize potential negative effects. Stakeholders that should be involved in the design, implementation, and monitoring of plastic clean-up technologies range from start-ups, entrepreneurs, device manufacturers, and distributors, to civil society, local governments, port authorities, and NGOs. We recommend development and implementation of EIA and LCAs, and standards for recording catch- and bycatch rates. Despite challenges posed, plastic clean-up technologies can provide important added value in terms of data collection and outreach to implement preventive efforts. Because of the role of these technologies to combat plastic pollution, these considerations should be included in the plastics treaty to secure their net benefit to the environment and society.

**Table 1:** Summary of Benefits and Risks Associated with Implementation of Clean-Up Technologies, as well as Policy Recommendations

Benefits of clean-up technologies	Risks of clean-up technologies	Policy recommendations
Removal of plastics and litter	Bycatch affecting ecosystems negatively	Predeployment evaluation of interaction with ecosystem components in time and space
		Documentation of bycatch
		Harmonization of bycatch calculations
		Bycatch limits
		Design requirements to limit bycatch
		Restrictions in time and space to limit bycatch
Removal of plastics and litter	Inappropriate technology for ecological, social, and economic setting	Holistic predeployment evaluation of site-specific ecological, social, and economic factors
Removal of plastics and litter	Malfunctioning of technology	Predeployment evaluation of risks related to malfunctioning and losses of technology
Removal of plastics and litter	Low cost-efficiency	Predeployment evaluation
		Documentation of catch-efficiency
		Harmonization of catch-efficiency calculations
Recovered litter enters the waste management system	High costs of disposal	Predeployment evaluation of waste management opportunities
	Low recycling potential	
	Immature technology for recycling and waste-to energy	Documentation of destiny of recovered litter
	Lack of waste management facilities	
	High transportation costs	
Higher environmental awareness	Clean-up technology seen as solution resulting in more littering and less focus on upstream solutions	Outreach programs of high quality focusing on real solutions that encourage critical thinking
Data on pollution levels and sources	Improper reporting and poor data quality	Harmonization of data collection protocols
	Poor quality of citizen-science data	Develop citizen science projects based on best practice recommendations
		Independent observers
		Protocols allow for identification of policy relevant items
Economic opportunities related to recovered plastics	No opportunities to safely pursue repurposing and recycling options	Predeployment evaluation of economic opportunities and potential social, ecological, and economic risks
		Development of business models

## Abbreviations

LCA: life cycle assessment
EIA: environmental impact assessment
ESM: environmentally sound management
CPUE: catch per unit effort
NGO: nongovernmental organization
